# Affectivism and the Emotional Elephant: How a Componential Approach Can Reconcile Opposing Theories to Serve the Future of Affective Sciences

**DOI:** 10.1007/s42761-024-00272-y

**Published:** 2024-09-20

**Authors:** Daniel Dukes, David Sander

**Affiliations:** 1 Swiss Center for Affective Sciences, University of Geneva, Geneva, Switzerland; 2 Department of Psychology, FPSE, University of Geneva, Geneva, Switzerland

**Keywords:** Affectivism, Componential approach, Future of affective science(s)

## Abstract

This article discusses how the *affectivism framework* and the *componential approach* to emotion may serve the future of affective sciences. A particular aim of the article is to show that an appraisal-based componential approach to emotion can help reconcile opposing theories. It begins by contextualizing the evolution of emotion science within the framework of affectivism, acknowledging that the significant epistemological differences between various theories have paradoxically spurred interest in studying emotion across various perspectives and disciplines. If affectivism is regarded as the pursuit of a deeper understanding of not only emotions and other affective processes but also cognitive and behavioral processes, then its success can be partly attributed to the existence of multiple approaches, allowing each discipline and perspective to advance using the most suitable theory and methodology. We contend that a componential approach reveals that the five principal theories of emotion have each focused on one of five components of emotion. Overall, based on the analysis of several articles published in the *special issue on the future of affective science,* we argue that affective scientists are well equipped not only to build a future in which conceptual and methodological tools will be used to test diverging hypotheses between competing theories but also to acknowledge and celebrate where such theories converge.

In their editorial introduction to the Special Issue (SI) on the *future of affective science*, Shiota et al. (2023) highlight the *war* between two theoretical *camps—*Basic Emotion Theory and Constructionist theory*—*and underscore the enduring significance of a third: Appraisal Theories of emotion. They conclude that Appraisal Theories, including the Component Process Model, “should be taken seriously in efforts to reconcile alternative theories and generate new options” (p. 431). After relating this interesting and at times provocative SI in terms of the era of affectivism, we address the challenge made to Appraisal Theories by endeavoring to demonstrate their potential reconciliatory and generative power through the componential approach.

Affectivism is the approach in which the inclusion of affective processes in models of behavior, mind, and brain “not only explains affective phenomena but, critically, further enhances the power to explain cognition and behavior” (Dukes et al., 2021, p. 816). Integrative and interdisciplinary from its inception, affectivism covers the trajectory (history, present, and future) of the affective sciences, and excellent examples abound in the current SI. Indeed, several of the papers perfectly illustrate the affectivist approach. For instance, Ferrer and Gillman’s (2023) case study advocates for the inclusion of affective processes in models of behavior change that have historically “largely focus[ed] on social cognitive determinants, omitting affective determinants or including them in a superficial way” (p. 586, see also Stussi et al., 2024). Simmons et al. (2023) provide another example by demonstrating how the National Institutes of Health (NIH) contribute to the rise of affectivism by increasingly supporting research on key, transversal themes of the affective sciences, including stress, positive emotions, and emotion regulation.

As pointed out by the editors, there are different and divergent theoretical and methodological approaches associated to affective sciences. While, ultimately, convergence across approaches might be expected and even encouraged (see Scherer, 2022), it is important to note that this lack of consensus has played an important part in its interdisciplinary appeal and growth. For example, in terms of the two major psychological theories of emotion cited by the editors, it is generally true that a basic emotion approach has been adopted in affective neuroscience and affective computing, while a constructionist approach has been prevalent in, for example, the study of the history of emotion and in anthropology. One of the enduring strengths of affectivism then has been that its object—emotion and other affective processes—has been studied from many different, competing, angles. In bringing the perspectives together, we argue here that a particularly unifying approach is the componential approach to emotion which can be defined as one that analyses the emergence of an emotion in terms of *its interacting constitutive components*. Although some componential approaches were proposed for instance in the nineteenth century already (e.g., McCosh, 1880; see Sander, 2022 for discussion), they mostly developed in the twentieth century and are explicitly formulated in influential versions of some of the major current theories of emotion: the Basic Emotion Theory (e.g., Matsumoto & Ekman, 2009, p. 69; Ekman, 1992; see Shiota, 2024), Psychological Constructionism (e.g., Russell, 2009, p. 125; see Russell, 2021), and Appraisal Theories (e.g., Scherer, 2005, p. 697; see Moors et al., 2013). In fact, a historical survey of competing definitions revealed one of the greatest commonalities to be that “emotion contains several important components” (Kleinginna & Kleinginna, 1981, p. 352). Although the exact number and nature of components is still up for debate as, consequently, are the number and types of possible interactions between them (see Lange & Zickfeld, 2021; Mauss et al., 2024; Sander, in press; Scherer, 2022), typically considered components are *emotion elicitation* (e.g., appraisal), *expression*, *autonomic activity*, *action tendencies*, and *feeling* (see Sander, in press). A componential approach then is an approach that takes into account several interacting components in explaining the nature and functions of emotion (see Fig. 1).

**Fig. 1:**
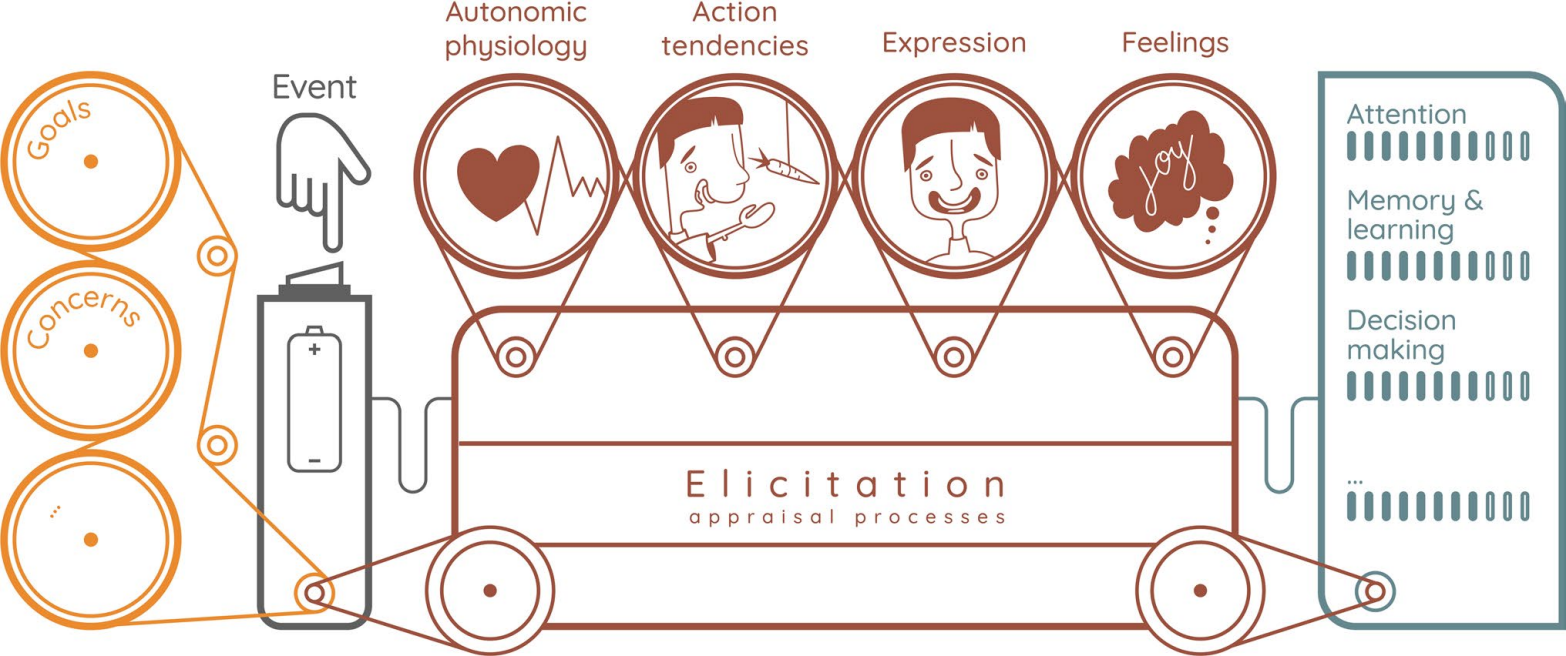
Illustration of a componential approach to emotion (from Pool & Sander, 2021). An event is appraised (elicitation) by the individual according to their current values concerns, and goals. This process triggers an emotion response involving several components: autonomic physiology, action tendency, expression, and feeling. All emotion components interact among themselves and modulate cognitive processes such as attention, memory, learning, and decision-making

We argue that much of the debate about which emotion theory is best occurs because emotion has several different components, and each major theory, while acknowledging the componential nature of emotion, centers on one of them. At this point, we cannot resist the temptation to refer to the parable of the blind men and the elephant (see also Russell and Barrett, 1999). Perhaps, just as in the parable of the blind men fighting over the nature of an elephant, each of the five major theories of emotion has one component as its prime focus: The role of *cognition in emotion-elicitation* is central to the Appraisal Theories, *expression* is central to Basic Emotion Theory, *feeling* is central to Constructionist theories, *action tendencies* to Motivational Theories, and *autonomic activity* to Bodily/Interoceptive Theories (for a more detailed analysis, see Sander, in press).

Most studies in affective science, for good reasons that relate to the theory, the research question, and/or to the method that is used, focus on one particular component. For example, *emotion elicitation* is the focus of several studies of the SI (Ferrer & Gillman, 2023, Kako et al., 2023, Lin et al., 2023, Shore et al., 2023, and Sikka & Gross, 2023). We note that the technological and methodological innovations described by Kako et al. (2023) would allow the use of advanced virtual reality settings, thereby increasing the relevance of stimuli for participants.

Cheong et al. (2023) introduce an interesting new toolbox for supporting facial *emotion expression* processing, while Kappas and Gratch (2023) demonstrate the broad and increasingly powerful contribution of affective computing to affectivism. Meanwhile, Cross et al. (2023) point out the danger of exclusively using facial expression recognition software, in particular when aiming to reliably recognize naturally occurring emotions; this is a good example of how useful it can be to bring converging evidence from other expressive channels (e.g., voice or posture) and other components (e.g., appraisal outcomes, action tendencies, psychophysiology or feeling) to correctly recognize the actual emotion (see Delplanque & Sander, 2021). Another component of emotion, *autonomic activity* is covered by Hoemann et al. (2023), Park et al. (2023) and Brady et al. (2023).

A relatively neglected component of emotion, *action tendencies*, deserves particular attention for the future of affective sciences. This neglect is somewhat reflected in the current SI as there are few papers that cover it. As Walle and Dukes (2023) point out in the SI, while valence is usually considered in terms of feeling, it is sometimes better considered in terms of action tendencies—approach (positive) and avoidance (negative). As Wood and Coan (2023, also in the SI) point out, it is often the commonalities in goals, bodies, and environment that lead to recognizable action tendencies.

One relatively renewed focus of attention among emotion researchers is that of how affective dynamics evolve in the *feeling* component of emotion. Given the obvious link between self-reports and language, new methodologies for analysis are suggested (Teoh et al., 2023; Tran et al., 2023) for deepening our understanding of language and well-being (Nook, 2023) and about how to get a more direct measure of feeling (Rocklin et al., 2023) are proposed with the SI.

While most of the SI papers focus on one component, three papers stand out as more explicit demonstrations of the potential utility of a componential approach. The first is the study by Abatista and Cova (2023) who found that self-transcendent emotions can be differentiated into at least two main different families by using the components of appraisals, bodily feelings, and action tendencies. The second is by Vishkin and Tamir (2023) who propose a useful conceptual tool to address many research questions concerning the components of emotion and their regulation. Indeed, in explaining how norms for emotions can apply to any of the components of the emotion response (e.g., you should smile, you should be happy), the authors point to a key variable that has the potential, through influencing appraisal and reappraisal, to increase the coherence among the various components of emotion.

But perhaps the most ambitious paper to feature in the SI in terms of theoretical perspective is also the one that, although perhaps inadvertently, best highlights the possibilities for the componential approach to reconcile opposing theories. Wood and Coan (2023) share the same objective as our commentary: exploring how certain perspectives can reconcile opposing theories to serve the future of affective sciences. The affectivism framework and the componential approach that we put forward as solutions are fully compatible and, even, potentially reinforced, by the solutions put forward by Wood and Coan (2023) in updating the terms of the debate in line with contemporary evolutionary biology. This perspective which no longer pits “nature vs nurture”, suggests instead that, “at the level of goal-directed behavior, emotions are relatively universal, discrete, and adaptive—one might say evolved—but also necessarily constructed through the body’s interaction with the environment” (p.443). Emotions are not only reactions to events that interact with our goals, but they are useful to pursue our goals. The dynamic systems framework that they propose views emotions as emergent attractors.

In terms of comparison with a componential approach, they explicitly state that their goal-centric approach is compatible with some appraisal theories of emotion and “with more recent characterizations of emotions as tracking progress toward (or away from) goals (Cunningham et al., 2013; Kron & Weksler, 2022)” (Wood & Coan, 2023, p. 446), pointing out that to classify an emotion based on one or other of the single components is to misunderstand how emotions emerge. A related similarity with the appraisal perspective to emotion is that some appraisal approaches have also proposed that emotions can be profitably explored with the help of nonlinear dynamic systems theory, given the potential role of appraisals in generating attractor basins (e.g., Sander et al., 2005; Scherer, 2000; see also Sacharin et al., 2012 for the role of hysteresis in emotion perception). In highlighting the key importance of goals (e.g., to mate, to play, to be safe), Wood and Coan propose a relational approach that is a biologically-based update of some functionalist perspectives advocated by scholars such as Frijda (e.g., Frijda, 1986) and Campos (e.g., Campos et al., 1994). And just as in those foundational texts, Wood and Coan highlight the importance of *goal relevance*—a phenomenon that appears to be accepted as vital to emotion elicitation, irrespective of the favored emotion theory (Wharton et al., 2021).

Just as it is fundamental to avoid *caricaturing* theoretical models in order to stay away from *a straw man fallacy*, Wood and Coan show that it is fundamental to avoid *caricaturing* constructs from specific disciplines (e.g., genetics) in order to stay away from wrongly formulated debates. For instance, a non-superficial understanding of genetics in biology may avoid caricaturing—and help moving beyond—the “Nature versus Nurture” debate. This is a challenge for the recent academic field of affective sciences because affective scientists who are typically trained in one discipline (e.g., psychology) need therefore to gain up-to-date knowledge from other disciplines (e.g., biology, philosophy or computer sciences). The future of the field therefore also relies on a truly multidisciplinary training of affective scientists.

In conclusion, reading the papers of the SI highlights that affective scientists are well equipped with conceptual and methodological tools to jointly build a future where points of divergence remain scientifically useful but where points of convergence are also acknowledged and celebrated. This commentary aimed at showing that both the *affectivism framework* and the *componential approach* provide the tools to serve the future of affective sciences.

## Additional Information

### Data Availability

No datasets were generated or analysed during the current study.
